# An alternative inhibitory avoidance task for studying hippocampus-dependent spatial aversive memory in mice

**DOI:** 10.1186/s13041-026-01308-z

**Published:** 2026-05-03

**Authors:** Haiyan Wang, Masanori Nomoto, Emi Murayama, Kaori Yamada-Nomoto, Kaoru Inokuchi

**Affiliations:** 1 Research Center for Idling Brain Science, University of Toyama, Toyama, 930-0194 Japan; 2 Department of Biochemistry, Graduate School of Medicine and Pharmaceutical Sciences, University of Toyama, Toyama, 930−0194 Japan; 3 CREST, Japan Science and Technology Agency (JST), University of Toyama, Toyama, Japan; 4 Japan Agency for Medical Research and Development (AMED), Tokyo, Japan

**Keywords:** Alternative inhibitory avoidance, Spatial aversive memory, Route choice, Hippocampus, Spatial navigation

## Abstract

In natural environments, animals must navigate to goals while avoiding　potential danger, making adaptive route choice crucial for survival. However, behavioral tasks for quantitatively evaluating avoidance through route choice during spatial navigation remain limited in mice, and the neural mechanisms underlying experience-dependent updating of route choice remain incompletely understood. Here, we established an air puff-based alternative inhibitory avoidance (AIA) task in mice to examine how aversive experience modifies a previously learned route preference. Water-restricted mice were first trained for 3 consecutive days to prefer a short path for obtaining water reward. They were then trained to avoid this preferred short path by receiving an air puff at its center when they passed through it. Mice that received only 3 air puffs showed lower avoidance behavior at the 6-h memory test. In contrast, mice that continued to receive air puffs until they rarely selected the short path during training showed significantly stronger avoidance at the 6-h test, and this avoidance was also observed at the 24-h test. We next examined whether hippocampal activity is required for retrieval of aversive memory in the AIA task. Chemogenetic suppression of hippocampal activity 30 min before the 6-h test impaired retrieval of aversive memory. Together, these results indicate that the AIA task provides a useful behavioral paradigm for assessing experience-dependent changes in route choice based on aversive events in mice, and that retrieval of this spatial aversive memory depends on hippocampal activity.

**Supplementary Information:**

The online version contains supplementary material available at 10.1186/s13041-026-01308-z.

## Introduction

In daily life, retrieval of aversive memory helps animals adapt their behavior using spatial and contextual information. For example, after encountering an aversive event along a familiar route, an animal may later detour around that location while still heading toward the same destination. This type of behavior suggests that aversive memory associated with a specific location can influence subsequent actions and route choice [1]. However, how animals alter later behavior and route choice after acquiring aversive memory for a specific location remains incompletely understood.

Contextual fear conditioning (CFC) and inhibitory avoidance (IA)/passive avoidance tasks have long been used to study aversive memory. In CFC, freezing is the principal behavioral readout, whereas in IA and passive avoidance tasks, memory is mainly evaluated by entry latency or response suppression [2–4]. These paradigms have provided important insights into aversive memory. However, they are not designed to directly assess how aversive memory associated with a specific location influences subsequent route choice while animals continue to move toward a goal.

By contrast, spatial arena tasks can assess spatial aversive learning during ongoing navigation. In particular, the rotating-arena active place avoidance task in mice is a well-established paradigm for hippocampus-dependent spatial avoidance learning [5]. However, its primary readout is avoidance of a dangerous zone rather than explicit choice between alternative routes leading to reward. Recent studies have further suggested that the hippocampus incorporates not only spatial information but also aversive or behaviorally relevant information during learning [6–8]. In rats, a related route-choice paradigm showed that aversive experience can alter preference for a previously rewarded path [9]. Nevertheless, behavioral paradigms remain limited in mice for quantitatively examining how aversive memory associated with a specific location influences later reward-directed route choice. To address this issue, we established an air puff (AP)-based alternative inhibitory avoidance task in mice.

## Results

**Fig. 1:**
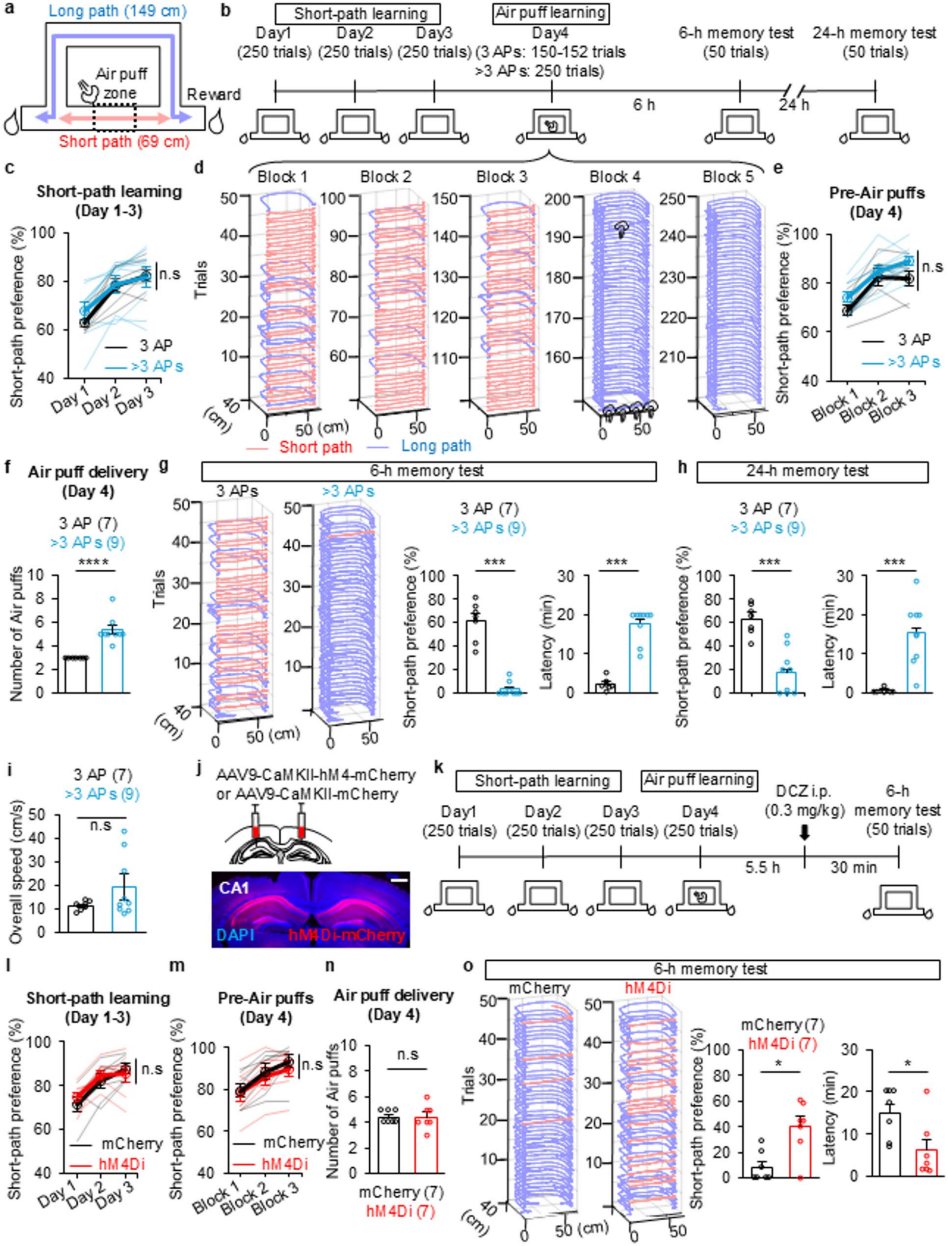
Alternative inhibitory avoidance behavior depends on aversive experience during training and on hippocampal activity during memory retrieval. **a** Schematic diagram for the alternative inhibitory avoidance (AIA) task. **b** Behavioral timeline for the learning and test phases. **c** Short-path preference during the initial learning phase across 3 days (RM two-way analysis of variance for day × group interaction, for group factor, *F*_(1,14)_ = 0.1199, *P* = 0.7343; for day factor, *F*_(2,28)_ = 55.88, *P* < 0.0001). **d** Representative mouse trajectories extracted by DeepLabCut during the air puff learning phase from > 3 APs group (50 trials per block). **e** Short-path preference before air puff delivery (RM two-way analysis of variance for block × group interaction, for group factor, *F*_(1,14)_ = 2.386, *P* = 0.1448; for block factor, *F*_(2,28)_ = 55.90, *P* < 0.0001). **f** Number of air puffs delivered on Day 4 in the 3 APs and > 3 APs groups (Mann-Whitney test, *P* < 0.0001). **g** Behavioral trajectories, short-path preference (Mann-Whitney test, *P* = 0.0002), and latency until first short-path choice (Mann-Whitney test, *P* = 0.0002) at the 6-h memory test, respectively. **h** Short-path preference (unpaired *t*-test, *P* = 0.0001) and latency until the first short-path choice (Mann-Whitney test, *P* = 0.0002) at the 24-h memory test. **i** Overall mean speed at 6-h test from two groups. Movie data sampled at 60 Hz was used to extract mice position and calculate trial-by-trial mean speed to compare the locomotion between two groups. The overall mean speed (cm/s) was obtained for each mouse by averaging across all trials (Mann-Whitney test, *P* = 0.2991). **j** Viral vectors used for hippocampal chemogenetic silencing and coronal section of the hippocampus with mCherry-expressing cells. All the animals incorporated as data showed similar expression patterns (Fig. S5). Scale bar, 500 μm. **k** Behavioral timeline for hippocampal silencing during 6-h memory test phase. **l** Short-path preference during the initial learning phase across 3 days (RM two-way analysis of variance for day × group interaction, for group factor, *F*_(1,12)_ = 0.1060, *P* = 0.7503; for day factor, *F*_(1.845, 22.14)_ = 23.73, *P* < 0.0001). **m** Short-path preference during the air puff learning phase (Day 4) (RM two-way analysis of variance for block × group interaction, for group factor, *F*_(1,14)_ = 0.6327, *P* = 0.4396; for block factor, *F*_(2,28)_ = 38.43, *P* < 0.0001). **n** Number of air puffs delivered on Day 4 in the mCherry and hM4Di groups (Mann-Whitney test, *P* = 0.9999). **o** Behavioral trajectories, short-path preference (Mann-Whitney test, *P* = 0.0140), and latency until the first short-path choice (unpaired *t*-test, *P* = 0.0223) at the 6-h memory test

To examine how aversive experience affects later route choice, mice were trained in an air puff-based alternative inhibitory avoidance (AIA) task in a rectangular maze (Fig. 1a and Fig. S1). Behavioral trajectories were extracted with DeepLabCut for subsequent analysis [10]. Water-restricted mice were first trained to acquire a stable preference for the short path to obtain water reward. They were then trained to avoid this preferred short path by receiving an air puff at its center when they passed through it (Fig. 1b and Figs. S2, S3).

During the initial spatial training phase, all mice developed a robust preference for the short path across 3 days, and this preference was comparable between groups later assigned to different aversive exposure conditions (Fig. 1c). Mice were then trained to avoid this previously preferred path by receiving an air puff at its center when they passed through it (Fig. 1d, e and Supplementary Movie 1). During this phase, the > 3 APs group received significantly more air puffs than the 3 APs group, which was immediately removed from maze after 3 APs, and continued training until they rarely selected the short path (Fig. 1f). Since it has been reported that early post-learning sleep is a critical time window for memory consolidation [11], mice were tested 6 h and 24 h later, following air puff learning, in the absence of aversive stimuli to assess aversive memory retrieval at an early post-learning consolidation phase [11] and a later memory retention stage, respectively (Fig. 1g, h). At both the 6-h and 24-h memory tests, the > 3 APs group showed significantly stronger avoidance than the 3 APs group, as indicated by lower short-path preference and longer latency to the first short-path choice (Fig. 1g, h and Supplementary Movie 2). To further address distinct patterns with air puff delivery, we compared the correlation between APs patterns and behavioral performance at 6-h and 24-h test phases (Additional file 4). Short-path performance did not show correlation with APs pattern (*P* > 0.05), but latency at 24-h test showed correlation with a pattern in which mice received multiple APs at a single trial (*P* = 0.0119) (Additional file 4). Trajectory-based locomotion speed analysis showed no significant difference between two groups in overall mean speed for task engagement (Fig. 1i), suggesting that behavioral difference is not attributable to generalized locomotor slowing. In addition, we compared the completion time for both long and short paths at 6-h test between two groups (Fig. S4). No significance was observed, suggesting that the observed behavioral difference is due to memory expression rather than reduced motivation (Fig. S4). These results indicate that the expression of aversive memory was influenced by the extent of aversive experience during training.

We next examined whether hippocampal activity is required for retrieval of aversive memory in this task. Mice received bilateral injections of virus vector expressing hM4Di-mCherry or mCherry in excitatory cells (Figs. 1j and S5), and hippocampal activity was suppressed by systemic injection of deschloroclozapine (DCZ) 30 min before the 6-h memory test (Fig. 1k). hM4Di-expressing mice performed comparably to control mice during both the spatial and air puff learning phases, with no differences in short-path preference learning or in the number of air puffs received (Fig. 1l–n, S6 and S7). In contrast, DCZ-treated hM4Di-expressing mice showed impaired aversive memory retrieval at the 6-h test, with higher short-path preference and shorter latency to the first short-path choice than control mice (Fig. 1o, S6 and S7). Together, these results suggest that later avoidance behavior is shaped by the extent of aversive experience during learning and depends on hippocampal activity during retrieval at the 6-h test.

## Discussion

Hippocampal cognitive map representations are thought to support flexible navigation in familiar environments, including movement toward reward-associated locations and avoidance of locations associated with aversive experience [12]. Previous rat study which applied similar route choice-based task showed that the route choice can be changed by the aversive experience [9]. However, the necessity of hippocampus for adaptive route choice was not examined.

In the present study, we established an air puff-based alternative inhibitory avoidance task in mice to examine how aversive memory associated with a specific location influences subsequent route choice during goal-directed behavior. Mice that continued to receive air puffs until they rarely selected the short path showed stronger avoidance, and this avoidance was still observed at the 24-h test. In addition, chemogenetic suppression of hippocampal activity impaired retrieval of aversive memory at the 6-h test. These findings indicate that the AIA task provides a mouse behavioral paradigm for assessing experience-dependent changes in route choice and that retrieval of this spatial aversive memory depends on hippocampal activity.

A key feature of this task is that it evaluates avoidance as an explicit change in route choice while animals continue to move toward rewards. This feature distinguishes it from conventional inhibitory avoidance tasks, in which memory is typically assessed by latency or response suppression, and from rotating-arena active place avoidance tasks, in which the main readout is avoidance of a punished zone rather than choice between alternative goal-directed routes [2–5]. Although this behavior may also reflect locomotor or motivational alterations, we did not detect a significant difference in either overall mean speed or completion time for both long and short paths at 6-h test phase between different air puff exposure conditions, suggesting that the observed avoidance behavior difference is not due to locomotion or motivation alterations. In addition, whereas a related route-choice paradigm has been reported in rats [9], the present task provides a mouse air puff-based paradigm that can be used to quantitatively assess aversive memory retrieval through route choice. Therefore, the AIA task may be useful for studying how aversive memory linked to a specific location modifies later route choice.

Another advantage of this task is its compatibility with continuous in vivo electrophysiological recording. Because aversive stimulation is delivered by air puff rather than foot shock, the task avoids electrical artifacts associated with shock-based paradigms and may facilitate analysis of hippocampal population activity during both online behavior and subsequent offline periods. This may be useful for examining hippocampal replay and preplay, which have been linked to fear-related memory retrieval and prospective organization of future trajectories [13–15]. In this context, the AIA task may provide a useful platform for investigating how hippocampal ensemble dynamics contribute to the updating and retrieval of spatial aversive memory.

## Supplementary Information

Below is the link to the electronic supplementary material.

Supplementary Material 1: Detailed Methods and Supplementary Figures

Supplementary Material 2: Data Source and Statistical Analysis

Supplementary Material 3: Data Source

Supplementary Material 4: Data Source and Statistical Analysis

Supplementary Material 5: Representative Movie for Air puff Learning Day

Supplementary Material 6: Representative Movie for the 6-h Memory Test

## Data Availability

No datasets were generated or analysed during the current study.

## Abbreviations

AIA: Alternative inhibitory avoidance
AP: Air puff
DCZ: Deschloroclozapine
DLC: DeepLabCut
RM: Repeated measures
