# Supplementary material for: An alternative inhibitory avoidance task for studying hippocampus-dependent spatial aversive memory in mice
Source: Mol Brain. 2026 May 3;19:47. doi: 10.1186/s13041-026-01308-z (PMC13281456; doi:10.1186/s13041-026-01308-z)
Supplement: Supplementary file 1 — Supplementary Material 1: Detailed Methods and Supplementary Figures [file 13041_2026_1308_MOESM1_ESM.docx]

**Additional file 1**

**An alternative inhibitory avoidance task for studying hippocampus-dependent spatial avoidance memory in mice**

**Haiyan Wang^1,2,3^, Masanori Nomoto^1,2,3,4*^, Emi Murayama^1,2,3^, Kaori Yamada-Nomoto^1,2,3^, and Kaoru Inokuchi^1,2,3*^**

*Corresponding authors.

Masanori Nomoto: [nomoto@med.u-toyama.ac.jp](mailto:nomoto@med.u-toyama.ac.jp)

Kaoru Inokuchi: [inokuchi@med.u-toyama.ac.jp](mailto:inokuchi@med.u-toyama.ac.jp)

**Methods**

**Mice**

Wild-type male C57BL/6J mice were obtained from Sankyo Labo Service Co. Inc. (Tokyo, Japan). Mice were maintained on a 12-h light/dark cycle at a controlled temperature (24 ± 3°C) and humidity (55 ± 5%) environment, with standard laboratory diet and tap water ad libitum. Mice used in all behavioral experiments were 12-20 weeks old. All behavioral experiments were conducted during the light phase. All procedures involving the use of animals complied with the guidelines of the National Institutes of Health and were approved by the Animal Care and Use Committee of the University of Toyama (Approval numbers: A2022MED-7, A2025MED-07).

**Viral vectors**

For chemogenetics experiment, the recombinant Adeno-Associated Virus (AAV) vectors encoding AAV9-CaMKII-hM4Di-mCherry (Titer: 8.87×10^14^ vg/mL) and AAV9-CaMKII-mCherry (Titer: ≥1×10^13^ vg/mL) after 5-fold dilution with phosphate-buffered saline (PBS) (T900; Takara Bio Inc., Japan) were used. AAV9-CaMKII-hM4Di-mCherry was obtained from Addgene (Viral preps #50475, Cambridge, MA). AAV9-CaMKII-mCherry was obtained from Addgene (Viral preps #114469, Cambridge, MA).

**DCZ administration**

Deschloroclozapine (DCZ) (HY-42110, MedChemExpress) with a purity of 99.93% was dissolved in dimethyl sulfoxide (DMSO) (049-07213, FUJIFILM Wako Pure Chemical) to prepare a stock solution (10 mg/mL), which was stored at -20°C. The stock solution was diluted with 0.9% saline to a final DMSO concentration of 0.3%. DCZ was injected intraperitoneally (i.p.) at a dose of 0.3 mg/kg 30 min before the 6-h memory retrieval test.

**Stereotactic surgery**

Mice (8-16 weeks old) were placed on a stereotactic apparatus after being anesthetized with a triple mixture of medetomidine, midazolam, and butorphanol which has been described previously (Reference 1). A heating pad was used to keep body temperature stable throughout the surgery procedure. Eye ointment was used to prevent eyes from drying.

For the chemogenetic inhibition experiment, two craniotomies (~1 mm diameter) were made on the top of the bilateral hippocampus, 500 nL of AAV9-CaMKII-hM4Di-mCherry or AAV9-CaMKII-mCherry viral vector was injected bilaterally into the hippocampus targeting the CA1 coordinates (AP: -2.0 from Bregma; ML: ± 1.4 mm; DV: -1.3 from Bregma) at a speed of 100 nL min^-1^. Viral injections were conducted with a 10 µL Hamilton syringe (Model 80030, Hamilton, USA) fitted with a glass needle filled with mineral oil. The syringe was attached to an IMS-20 motorized microinjector (Narishige, Japan) for automated delivery.

After surgery, atipamezole (Antisedan, Nippon Zenyaku Kyogo Co., Japan), an antagonist of medetomidine, was injected at a dose of 1.5 mg/kg to help mice recover from sedation. In addition, mice were allowed to recover in their home cage for weeks before subsequent experiments.

**Alternative inhibitory avoidance**

*Arena setup:*

All behavioral experiments were conducted in a soundproof room in a rectangular maze (69 cm length × 40 cm width × 27 cm height). The maze consisted of acrylic-panel tracks (7 cm wide) forming both short and long routes. Two lick ports were located at the corners of the 69-cm side (with each lick port extending 8 cm outward). Mice could obtain rewards via either a short or a long route: the short path involved passing only the 69-cm side of the maze, whereas the long path required mice to reach the rewards by traversing the remaining three sides, corresponding to a total path length of 149 cm. To facilitate discrimination of this short path, black vertical tape stripes were affixed to the side walls along this route, providing distinct visual cues. The short path also contained two small floor segments made of identical material, forming a “step-down” area analogous to that used in classical inhibitory avoidance paradigms. A beam-type infrared (IR) sensor (EX-Z12FA, Panasonic Industry Co., Ltd.) was positioned at the midpoint of the short path. The sensor and other custom devices were connected to an RX8 Multi-I/O Processor (Tucker-Davis Technologies [TDT], USA), and TTL pulses were used to build a closed-loop system for behavioral control and task implementation. During the air puff learning session, air puff delivery was triggered when the mouse interrupted the IR beam at the midpoint of the short path. All behavioral phases were recorded using a multi-camera setup and AG-desktop recorder software (T. Ishii, Japan) at 60 Hz, and later mouse trajectories were extracted offline by DeepLabCut (DLC) (Reference 2). During behavioral tasks, an infrared LED was implemented to synchronize timestamps across events (for example, the timing of left/right reward delivery).

*Handling:*

Prior to behavioral tasks, mice were handled daily for 1 consecutive week to habituate them to the experimenter and minimize stress-related confounds during behavioral tasks. During handling, mice were gently picked up and allowed to explore the experimenter’s hands for approximately 5-10 min per day.

*Water restriction:*

Five days before the beginning of tasks, mice were water-restricted. Water (1.0-1.5 mL) was provided daily to maintain their body weight between 80-85% of their original weight to keep their motivation for task engagement.

*Short-path learning:*

Mice underwent spatial training for 3 consecutive days to learn a preference for the short path to get water rewards. Training consisted of a single session per day. At the start of each session, mice were gently placed in a corner of this rectangular maze away from the lick ports and allowed to freely explore the environment. A trial was triggered only when the mouse visited the left lick port, which served as the initial rewarded port. If the mouse visited the right lick port before trial initiation, no reward was delivered. Following trial initiation, mice were required to alternate between the two lick ports to obtain water rewards. After mice obtained a reward from one port, water availability at that port was terminated, and mice had to navigate to the other port for the next reward. Only a single reward was available at each port per trial. Each daily session consisted of 250 reward deliveries (125 per lick port, 250 trials), with each water reward delivered for 3 ms, corresponding to approximately 2-3 μL of sucrose water. After 3 days of spatial training, mice exhibited a short-path preference exceeding 75%.

*Air puff learning:*

Following short-path learning for 3 days, mice underwent an air puff learning phase. During the initial 150 trials, mice were allowed to obtain water rewards by alternately visiting the two lick ports, as in the spatial training phase. If mice showed a high short-path preference (>70%) during the 50 trials immediately before air puff delivery, air puff stimulation was delivered from the 151st trial when the mouse interrupted the IR beam at the midpoint of the short path. Air puff stimulation was delivered at 0.4 MPa using an air compressor (PROFIX NITRO-COMP Nitro Comp V2 Oil-less Air Compressor, Raywood, Japan).

To manipulate the extent of aversive experience, mice were assigned to different air puff exposure conditions. In the 3 APs group, mice were immediately removed from the maze after receiving 3 air puff stimuli, with an average of 150.7 ± 0.29 trials on Day 4 (Additional file 3). In the >3 APs group, mice continued training until completion of 250 trials, and air puff delivery was limited to a maximum of 10 stimuli and was presented during subsequent trials whenever the mouse traversed the short path. Under this condition, mice received an average of 5.3 ± 0.37 air puffs (Additional file 2). Animals that failed to meet the criteria were excluded from further experiments. To standardize behavioral evaluation, mice that remained immobile for more than 5 min during the task were considered to show insufficient task engagement and were excluded from continued task performance analysis.

*Memory retrieval test:*

Following the air puff learning phase, mice were gently picked up, returned to their home cages and allowed to rest or sleep in a quiet and familiar environment. Memory retrieval was assessed 6 h and 24 h later using the same maze configuration as during learning. During the memory retrieval tests, mice were allowed to freely navigate the maze and obtain water rewards under the original alternation rule between left and right ports. However, no air puff was delivered even if mice chose the short path. Behavioral performance during the tests was used to evaluate memory expressions of spatial aversive memory, including the short-path preference and latency until first short-path choice. The arena was cleaned using 70% ethanol after each subject and session.

*Chemogenetic inhibition experiment*:

For the chemogenetic inhibition experiment, hM4Di-expressing mice received intraperitoneal injections of DCZ (0.3 mg/kg) 30 min before the 6-h memory test, whereas mCherry-expressing mice received same DCZ injection as the control group.

**Behavior analysis**

All behavioral phases were recorded using the overhead multi-camera setup and AG-desktop recorder software (T. Ishii, Japan) at 60 Hz and were analyzed offline. For synchronization, infrared LED signals recorded during the behavioral task were aligned with timestamp signals generated by the TDT system. Mouse position was tracked using DLC, and custom MATLAB scripts were used for subsequent timestamp alignment and behavioral quantification. For each trial, path choice was classified as either short or long based on the spatial location of the mouse immediately prior to reward acquisition.

Short-path preference was calculated as the percentage of the trials that mice chose the short path over all trials during the experimental periods. In addition, the latency to the first short-path choice was quantified as the time elapsed until the mouse first chose the short path during the test phase. The equation for short-path preference was as follows:

$Short-path preference (\%) = 100 \times(trials with short-path choice / total trials)$A larger percentage change indicates greater preference for the short path over the long path during behavioral tasks, whereas a lower percentage indicates stronger avoidance of the short paths after air puff delivery.

**Immunohistochemistry and Microscopy imaging**

Following completion of behavioral experiments, mice were deeply anesthetized and transcardially perfused with phosphate-buffered saline (PBS) (pH 7.4), followed by 4% paraformaldehyde (PFA). Brain samples were carefully collected, post-fixed overnight in 4% PFA. The subsequent day, brains were cryoprotected in 25% sucrose in PBS at 4°C for 48 hours. After cryoprotection, brains were gently dried, frozen in dry ice powder, and stored at -80°C. Coronal brain sections (40 μm thickness) were prepared by a cryostat (Leica CM 3050) and processed using a free-floating protocol. As the viral constructs expressed fluorescent reporters, no additional immunostaining was performed. Sections were collected and immersed in a 12-well cell culture plate (Corning, NY, USA) in room temperature. For native nuclear staining, sections were counterstained with DAPI (2.5 μg/mL, Roche Diagnostics, 10236276001) for 20 min at room temperature and washed 3 times with PBS at 5-min intervals and then mounted on glass slides using Immu-Mount (9990402, Thermo Fisher Scientific Inc.).

Histological images were acquired using a Keyence fluorescence microscope (BIO-REVO, KEYENCE, Japan) with a Plan-Apochromat 4× objective lens. Viral expression and injection sites were verified by native fluorescence in coronal brain sections (Fig. S4). Only animals with correct and confined viral expression within the target region were included in the final behavioral analyses. Animals with mistargeted injections were excluded from analysis.

**Statistical analysis**

Statistical analyses were performed using GraphPad Prism 9. Normality of data distribution was assessed using the Shapiro-Wilk test. For comparisons between two independent groups, an unpaired Student’s *t*-test was used when the data were normally distributed, whereas the Mann-Whitney *U* test was applied when normality assumptions were not met. Repeated-measures (RM) ANOVA were applied for multiple learning days and across blocks comparisons. The statistical detailed information is described in figure legends and table in “Additional file 2”. Quantitative data are presented as mean ± SEM unless otherwise stated. Differences were considered statistically significant at *P* < 0.05.

**Supplementary Figures**


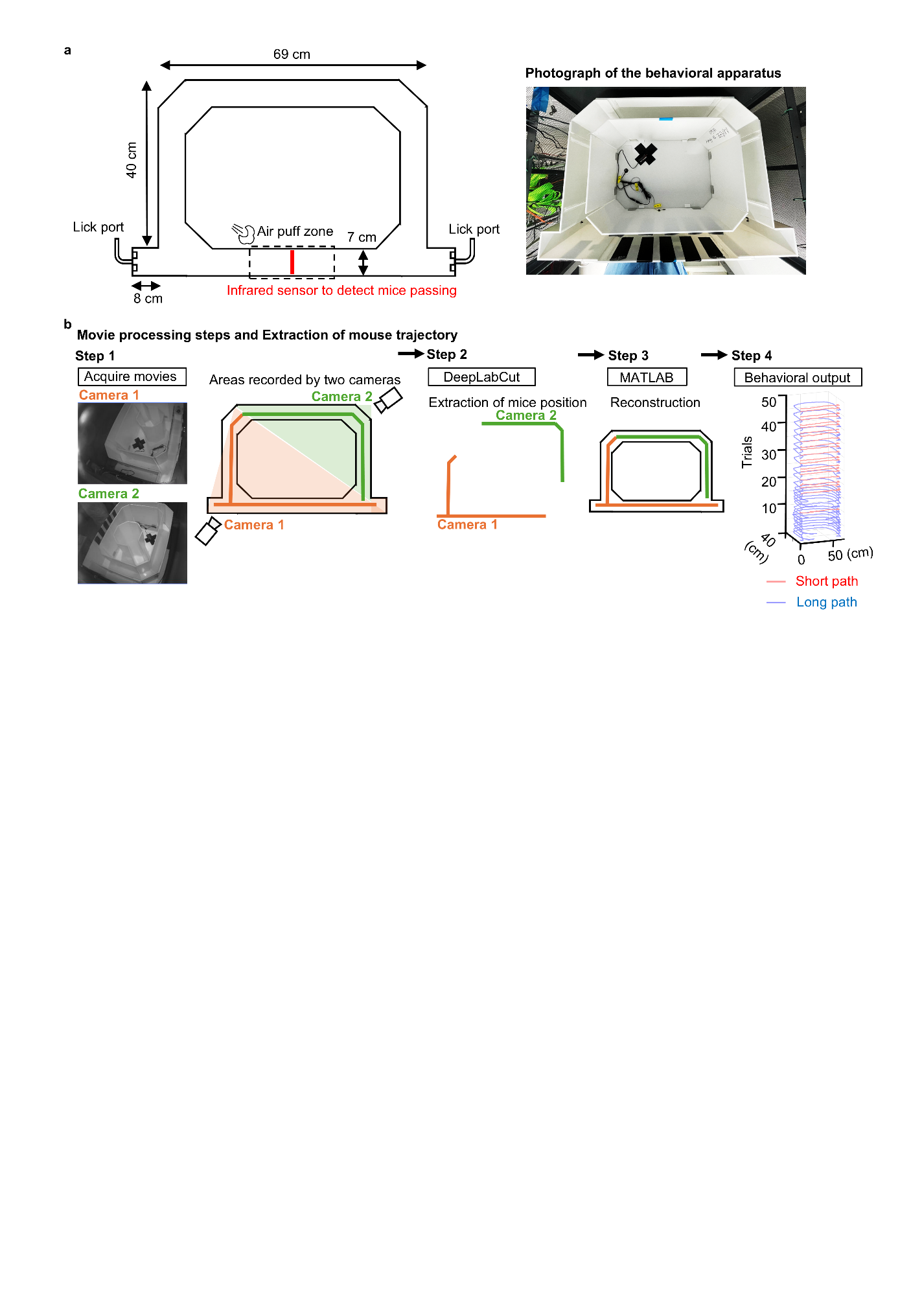


**Fig. S1 Behavioral apparatus and task configuration for AIA task.** **a** Schematic illustration of the rectangular arena (69 × 40 cm). Two water ports were positioned at the two corners along 69-cm side. The side directly connecting the two ports was designated as the short path, whereas the alternative route around the other three sides was designated as the long path. **b** Movie processing steps. Step 1: acquire movies by two cameras (camera 1 and camera 2) from different views of maze (indicated with light orange and green for two cameras respectively). Step 2: extract mice position using DeepLabCut. Step 3: reconstruct mice position to align with the whole rectangular maze. Step 4: behavioral performance output with red line for “short path” while blue line for “long path”.


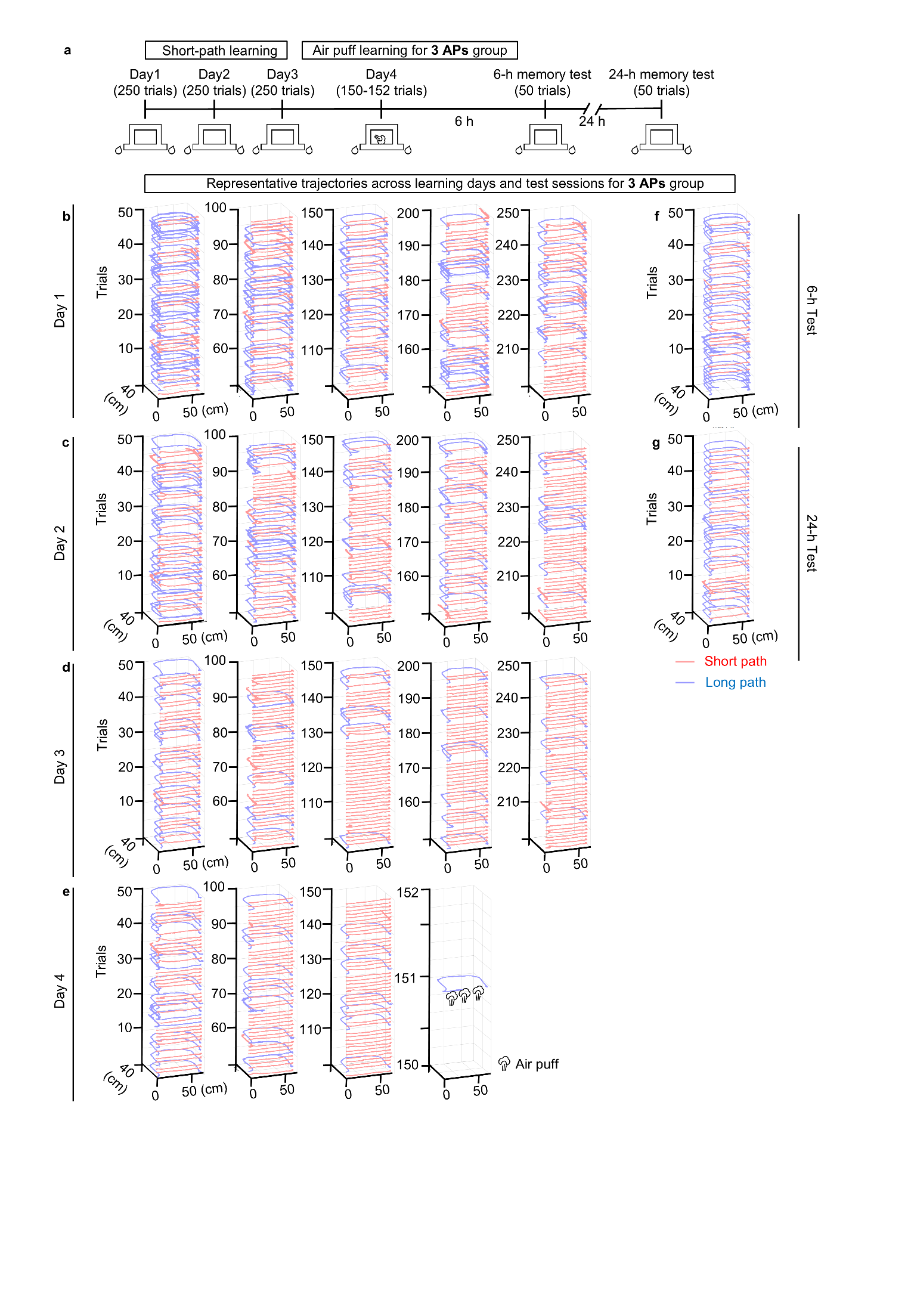


**Fig. S2 Representative mouse trajectories across learning and test phases for 3 APs group. a** Behavioral timeline for AIA task. **b-d** Representative mouse trajectories from 3 APs group on short-path learning Day1 **(b)**, Day 2 **(c)**, and Day 3 **(d)** (50 trials per block). **e** Representative trajectories on air puff learning phase (Day 4), this mouse received all three air puffs at 151^st^ trial. **f-g** Representative trajectories at 6-h memory test **(f)** and 24-h memory test **(g)**.


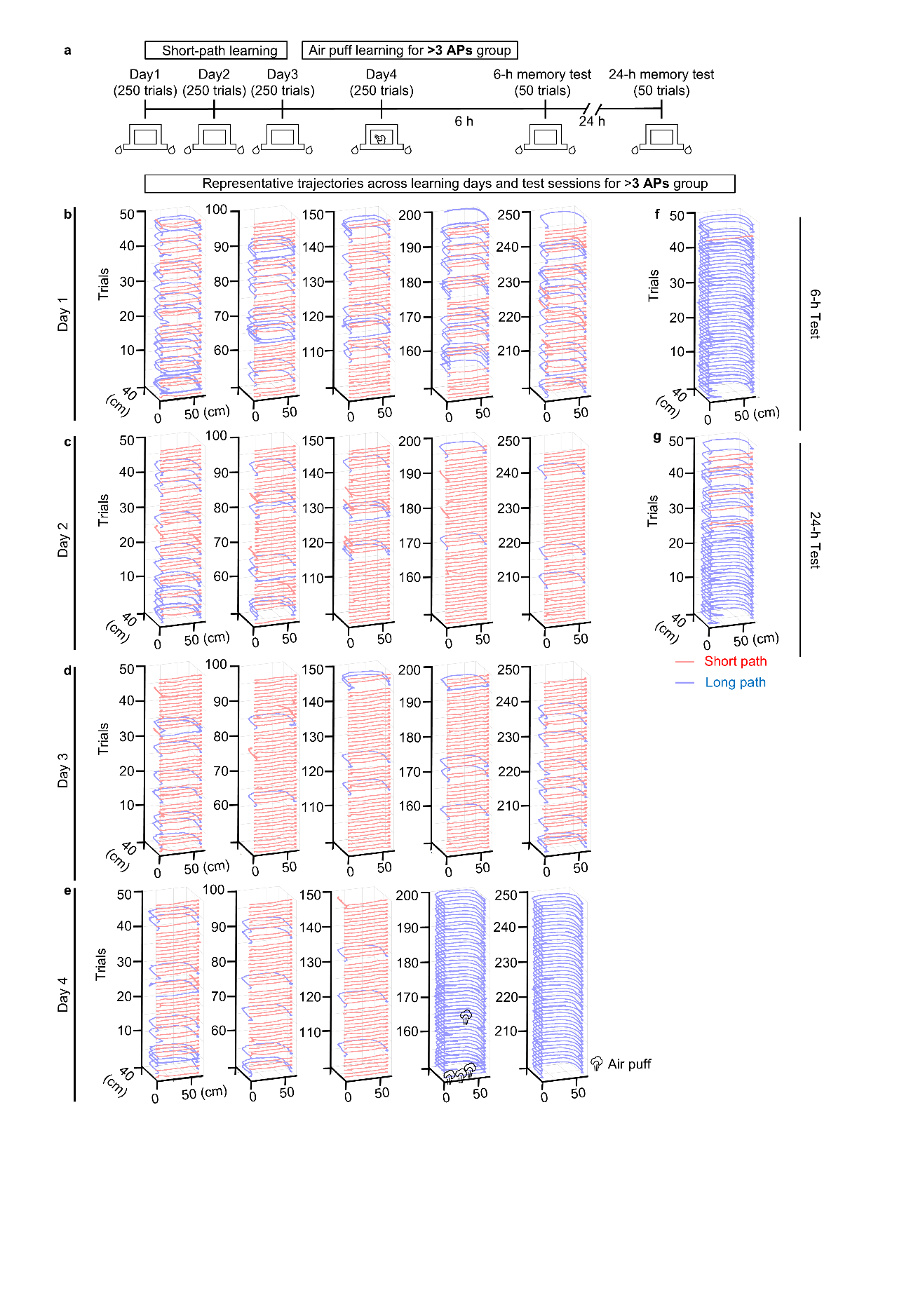


**Fig. S3 Representative mouse trajectories across learning and test phases for >3 APs group. a** Behavioral timeline for AIA task. **b-d** Representative mouse trajectories from >3 APs group on short-path learning Day1 **(b)**, Day 2 **(c)**, and Day 3 **(d)** (50 trials per block). **e** Representative trajectories on air puff learning phase (Day 4), this mouse received total four air puffs at 151^st^, 152^nd^, 154^th^, and 165^th^ trial. **f-g** Representative trajectories at 6-h memory test **(f)** and 24-h memory test **(g)**.


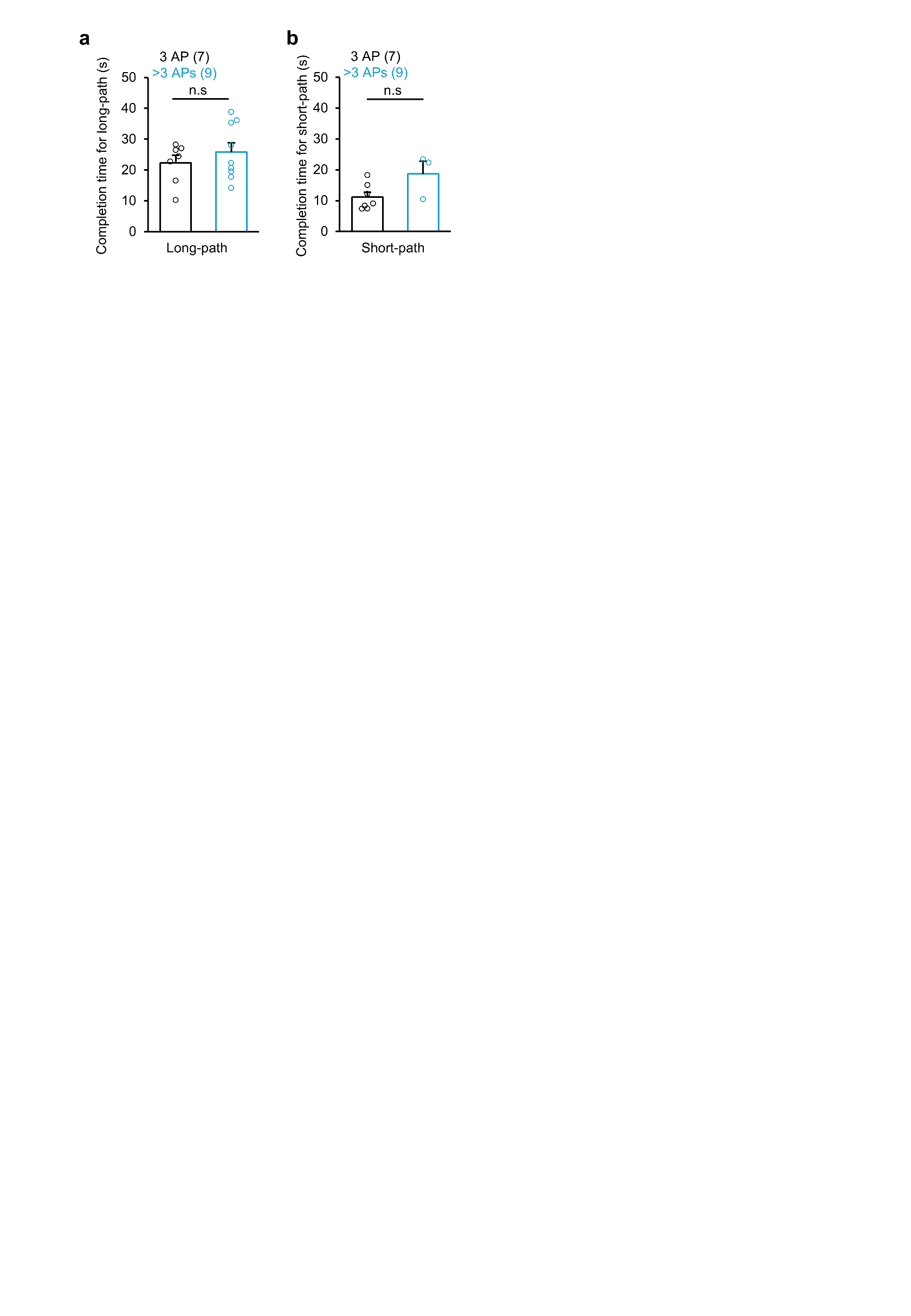


**Fig. S4 Completion time for both long and short paths at 6-h memory test to assess motivation between different air puff exposure conditions. a** Completion time for long-path between two groups (Unpaired t-test, *P* = 0.3776). **b** Completion time for short-path between two groups (Unpaired t-test, *P* = 0.1987). Among >3 APs group mice, because only 3 out of 9 mice showed short-path choice, there were only 3 dots representing 3 mice in the graph.


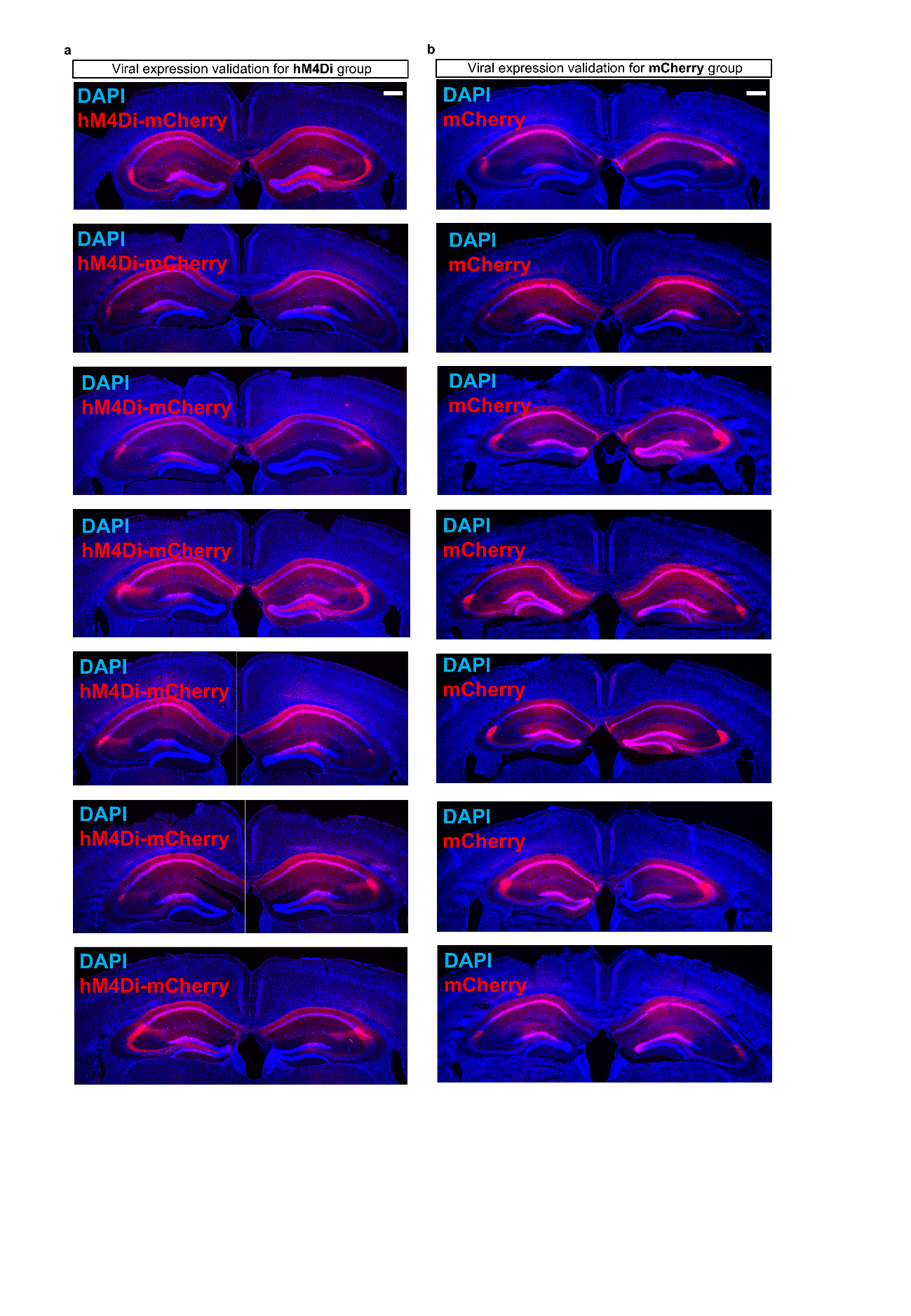
**Fig. S5 Viral expression validation for both hM4Di-expressing and mCherry-expressing mice incorporated as data. a-b** Coronal immunohistochemistry staining for hM4Di-expressing mice **(a)** and mCherry-expressing mice **(b)**. Scale bar, 500 μm


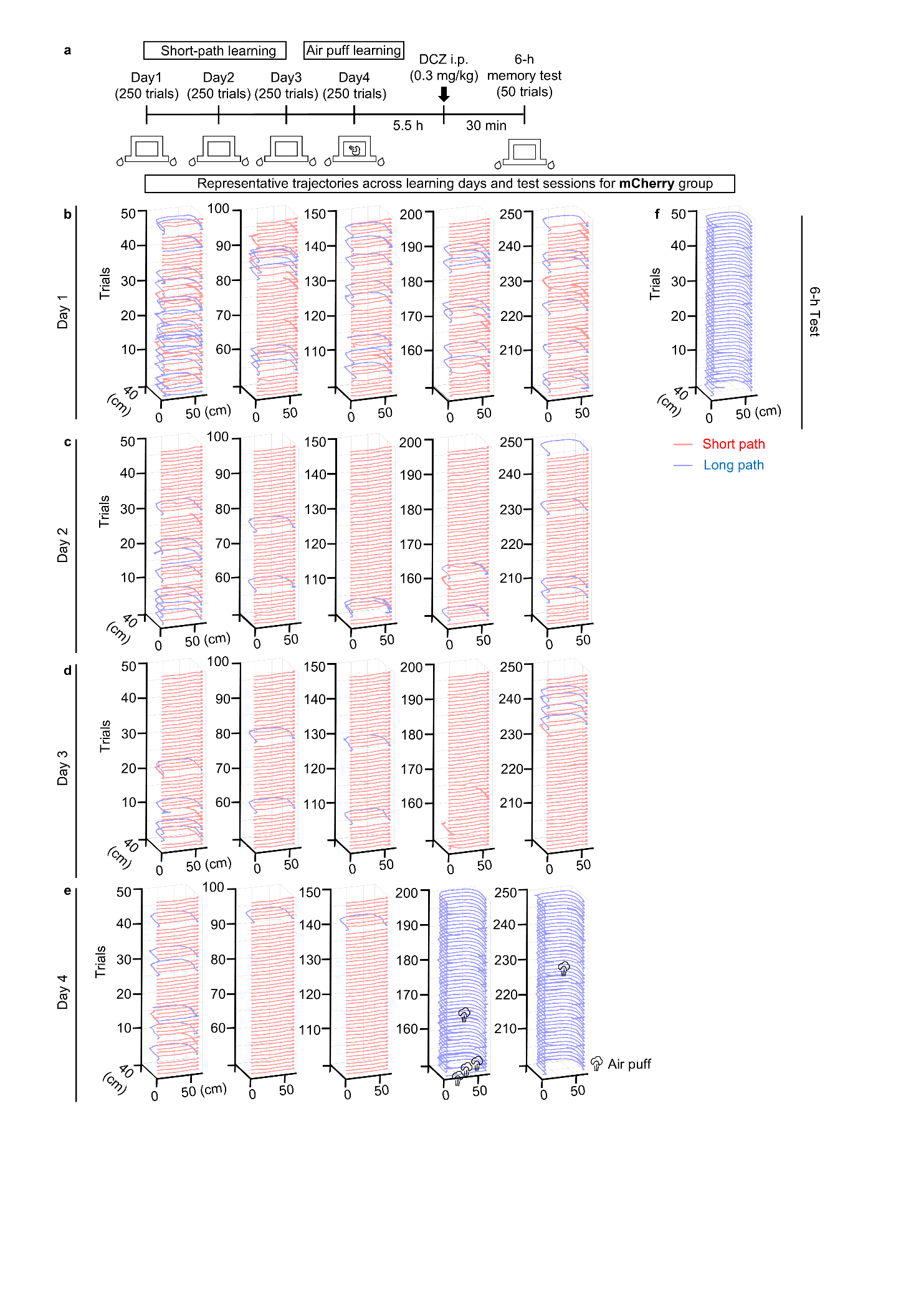


**Fig. S6 Representative mouse trajectories across learning and 6-h test for mCherry group. a** Behavioral timeline for AIA task. **b-d** Representative mouse trajectories from mCherry group on short path learning Day1 **(b)**, Day 2 **(c)**, and Day 3 **(d)** (50 trials per block). **e** Representative trajectories during air puff learning phase (Day 4), this mouse received total five air puffs at 151^st^, 152^nd^, 153^rd^, 167^th^, and 229^th^ trial. **f** Representative trajectories at 6-h memory test.


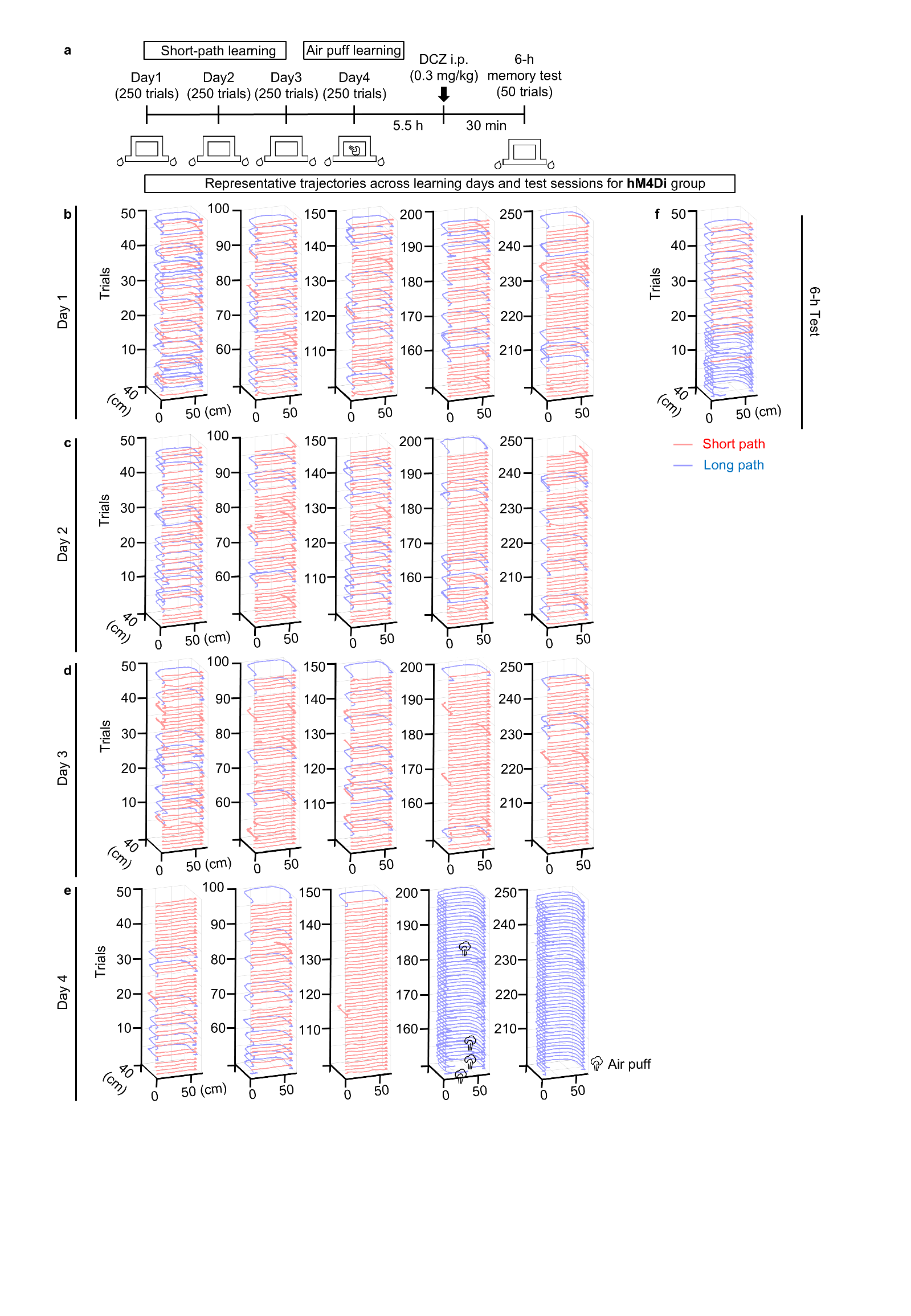


**Fig. S7 Representative mouse trajectories across learning and 6-h test for hM4Di group. a** Behavioral timeline for AIA task. **b-d** Representative mouse trajectories from hM4Di group on short path learning Day1 **(b)**, Day 2 **(c)**, and Day 3 **(d)** (50 trials per block). **e** Representative trajectories during air puff learning phase (Day 4), this mouse received total four air puffs at 151^st^, 152^nd^, 156^th^, and 183^rd^ trial. **f** Representative trajectories at 6-h memory test.

**Reference**

1. Nomoto M, Murayama E, Ohno S, Okubo-Suzuki R, Muramatsu S-i and Inokuchi K. Hippocampus as a sorter and reverberatory integrator of sensory inputs. Nat Commun. 2022;13:7413.

2. Mathis A, Mamidanna P, Cury KM, Abe T, Murthy VN, Mathis MW and Bethge M. DeepLabCut: markerless pose estimation of user-defined body parts with deep learning. Nat Neurosci. 2018;21:1281–1289.
